# Using Small-Scale Studies to Prioritize Threats and Guide Recovery of a Rare Hemiparasitic Plant: *Cordylanthus rigidus* ssp. *littoralis*

**DOI:** 10.1371/journal.pone.0008892

**Published:** 2010-01-26

**Authors:** Sean M. Watts, Melissa M. Uhl, Stephen P. Maurano, Erin E. Nuccio

**Affiliations:** 1 Environmental Studies Institute, Santa Clara University, Santa Clara, California, United States of America; 2 Department of Plant and Microbial Biology, University of California, Berkeley, California, United States of America; University of California, Berkeley, United States of America

## Abstract

**Background:**

Recovering endangered species would benefit from identifying and ranking of the factors that threaten them. Simply managing for multiple positive influences will often aid in recovery; however, the relative impacts of multiple threats and/or interactions among them are not always predictable. We used a series of experiments and quantitative observational studies to examine the importance of five potential limiting factors to the abundance of a state-listed endangered hemiparasitic annual forb, *Cordylanthus rigidus* ssp. *littoralis* (*C.r.l.*, California, USA): host availability, mammalian herbivores, insect seed predators, fire suppression, and exotic species. While this initial assessment is certainly not a complete list, these factors stem from direct observation and can inform provisional recommendations for management and further research.

**Methodology and Principal Findings:**

Studies were conducted at five sites and included assessments of the influence of host availability, exotic species, exclusion of mammalian herbivores and insect seed predators on *C.r.l.* productivity, and simulated effects of fire on seed germination. *C.r.l.* was limited by multiple threats: individuals with access to host species produced up to three times more inflorescences than those lacking hosts, mammalian herbivory reduced *C.r.l.* size and fecundity by more than 50% and moth larvae reduced seed production by up to 40%. Litter deposition and competition from exotic plant species also appears to work in conjunction with other factors to limit *C.r.l.* throughout its life cycle.

**Conclusions and Significance:**

The work reported here highlights the contribution that a series of small-scale studies can make to conservation and restoration. Taken as a whole, the results can be used immediately to inform current management and species recovery strategies. Recovery of *C.r.l.* will require management that addresses competition with exotic plant species, herbivore pressure, and availability of preferred host species.

## Introduction

Endangered species recovery relies on assumptions about limiting factors that are preventing recovery of imperiled populations. Tests of these assumptions are rarely reported in peer-reviewed journals [1]. Instead, threat analyses and corresponding management plans tend to be based on unpublished data and expert opinion, without well-documented field studies [2], [3].

Government agencies, ecological consultants, and reserve managers often conduct quantitative studies and possess a wealth of autecological data; however, much of this research remains inaccessible outside of these organizations. Meta-analyses drawn from these reports, e.g. the United States Department of the Interior, could provide practical resources for conservation and restoration practitioners. Absent such a meta-analysis, it is our opinion that conservation would be better served by the combined conclusions from rapid quantitative studies of the factors limiting particular species. The results from such studies could be compared with expert opinion and could act as a reference to help direct further research and to identify strategies to conserve, restore, and manage small populations (e.g. [4]).

Using quantitative studies to guide management has its own limitations. These include logistical constraints that result in narrow spatial and temporal scale experiments that may not allow for extrapolation to other geographic areas or may not encompass long term ecosystem dynamics ([5]). In addition, the multifactorial experiments that might best parse the complex threats to endangered species may be prohibitively expensive or result in unacceptable mortality of the target species. However, iterative and systematic experiments and observational studies can help direct attention to the most likely threats in complex systems, while also testing the effectiveness or unintended consequences of proposed management strategies (e.g. [6], [7]).

Here we apply an experimental and quantitative observational approach to identifying the factors that prevent the recovery of a parasitic plant that is considered endangered by the State of California. Rare parasitic plants face special problems when it comes to recovery because, in addition to basic habitat requirements, they must also locate suitable host plants from which to obtain water and nutrients [8]. The plant we studied is the hemiparasitic annual forb: seaside bird's beak, *Cordylanthus rigidus* ssp. *littoralis* ((Ferris) Chuang and Heckard, Orobanchaceae, formerly classified in Scrophulariaceae; hereafter *C.r.l.*). Of the 220 rare, threatened, or endangered species and subspecies in California, six are in the hemiparasitic genus *Cordylanthus* and seven are in the confamilial genus *Castilleja* ([9]).

Rabinowitz ([10]) identified seven forms of rarity in plants by considering geographic range (narrow – wide), local abundance (high – low), and habitat specificity (narrow – wide). Following this typology, *C.r.l.* is locally abundant over a large geographic range, with two population centers almost 200 miles apart and extending over 2° of latitude. However, *C.r.l.* has narrow habitat requirements: it is found only in the frequently disturbed sandy soils of stabilized dunes and further restricted by the availability of hosts. The recovery of *C.r.l.* is therefore tightly linked to the abundance and distribution of coastal woodlands and shrublands; landscapes that are becoming increasingly rare in the face of human development. The large geographic range of *C.r.l.* is encouraging; however, the isolation between its northern and southern populations suggests that they could be relictual and thus precarious.

Based on field observations and the natural history of this species, we focused on five potential limits to *C.r.l.* abundance:

1. *Host availability. Hemi*parasites retain some photosynthetic ability, but also receive water and nutrients from their hosts via haustoria (see supporting information Figure S1). Because *C.r.l.* are shallow-rooted annuals that flower toward the end of a long dry season, deep-rooted woody perennials are likely important hosts for *C.r.l.* [11]. *C.r.l.* in close proximity to shrubs appear to have greater survivorship and to be more productive than those growing in clearings.
2. *Mammalian herbivores.* Abnormally high rates of mammalian herbivory are common in reserves near developed areas, due to a lack of large predators and changes in habitat structure [12]. Deer, in particular, have greatly increased in abundance worldwide, and overbrowsing has cascading effects on vegetation dynamics and composition [13]. At the University of California, Fort Ord Natural Reserve, where we conducted our studies, abnormally heavy deer browsing limits population viability of *Ceanothus cuneatus* var. *rigidus* (Rhamnaceae), a shrub species of special concern [14]. Because a large proportion of *C.r.l.* stems were clipped at this site, we speculated that mammalian herbivores could exert a major effect on *C.r.l.* populations.
3. *Insect seed predators.* Exclusion of inflorescence-feeding insects promoted lifetime fitness even in a plant considered to be coevolved with its natural enemies [15]. Having observed moth larvae (*Clepsis* sp, Tortricidae) feeding in *C.r.l.* inflorescences, we assessed reproductive losses to these pre-dispersal seed predators.
4. *Fire suppression.* Fire suppression in Mediterranean-climates has changed the trajectory of many plant communities, threatening some natives that rely on post-fire habitats ([16]). The Monterey Bay area was subjected to frequent (yearly) intentional burning by Native Americans [17], but aerial photographs indicate that the Fort Ord area has not burned for at least 60 years [14]. We are not aware of previous work on fire-dependency in *C.r.l.*, but it is found in fire-adapted closed-cone pine forest and chaparral, suggesting that fire suppression might limit germination.
5. *Competition from exotic species (esp. annual grasses).* Exotic annual species have so thoroughly invaded California grasslands that they now define the “California annual system” [18]. Annual grasses in particular have long been recognized as strong competitors against both herbaceous and woody species [19]. Exotic grasses compete directly with native annual species through high germination densities and efficient resource use (e.g. water, light, nutrients), but also through the microclimate effects of high litter accumulation [19]. Although the phenology of *C.r.l.* minimizes competition during the summer drought season, February germination puts *C.r.l.* in direct competition with rapidly growing exotic winter annuals that often germinate in late November.

Although there are many potential factors we did not include, our assessment is based on field observations and represents information that is not often taken into account prior to initiating recovery programs. Ultimately studies like these could be compiled in a centralized database of research on endangered species- a widely recognized need by reviewers assessing recovery plans [20]. Such a database could be used by practitioners to weigh the potential impacts of specific management strategies and to highlight gaps in our knowledge about particular species.

## Materials and Methods

### Ethics Statement

Research on *Cordylanthus rigidus* ssp. *littoralis* was conducted under California Department of Fish and Game research permit #04-10-RP. Work at the University of California Fort Ord Natural Reserve (and associated Monterey Bay Education, Science, and Technology Center) was conducted under research application index number 6420 (Project Title: Watts_FONR Cordylanthus Restoration).

### Study Species and Site Description

*Cordylanthus rigidus* ssp. *littoralis* is a hemiparasitic annual forb with a low, branching habit and both axillary and terminal inflorescences. *C.r.l.* is patchily distributed in two areas of Monterey County and about 10 isolated sites in Santa Barbara County, California [21]. More detail is available as supporting information; see Site and Species Description S1.

We studied *C.r.l.* at maritime chaparral sites on a former military base (Fort Ord) 6.5 km northeast of Monterey, CA (21–58m elevation). The area has a Mediterranean-type climate, with late fall and winter rain and dry, but cool, foggy summers. Although year-to-year variation is considerable, *C.r.l.* in Monterey County generally germinate in mid-February and begin flowering in mid/late-July through August before senescing by mid-September (*pers obs*). Most other annual plant species have senesced by mid-June at these locations.

We investigated the influence of host proximity, mammalian herbivory, and insect seed predation within two naturally occurring populations of *C.r.l*: 1) the “FONR” population is roughly 4 km from the coast within the 242 ha University of California, Santa Cruz, Fort Ord Natural Reserve, 2) the “MBEST” population is on the property of the UCSC Monterey Bay Education, Science, and Technology Center and 1 km further inland (east) from the FONR population (Figure S2). In contrast to the FONR site, MBEST is not fenced, and is bounded on three sides by roads. UC FONR management conducted complete population surveys between 2001 and 2008; the MBEST population had consistently more individuals than the FONR population (these data are presented in supporting information; see Figure S3). To investigate potential limitations of fire suppression, exotic species and mammalian herbivory to the establishment of new populations, we used three additional sites within the Fort Ord Natural Reserve that did not include naturally occurring *C.r.l.*, and represented contrasting soil conditions and exotic species dominance (referred to hereafter as FONR “new”; see supporting information Figure S2: Sites C, D, and E). Studies were conducted over five years (2003–2007). Parenthetical statements following each subheading below indicate the year(s) and site(s) for each experiment (see Table 1 for a list of studies by site).

**Table 1:** Study descriptions by site and year.

Year	Site	Unit^a^	N b	Study Title	Main Factor/Treatment
2003	FONR	Indiv.	85 c	*Exotic species*	Exotic species litter depth & cover
2003	MBEST	Indiv.	35 c	*Exotic species*	Exotic species litter depth & cover
2004	MBEST	Indiv.	65 c	*Exotic species*	Exotic species litter depth & cover
2004	FONR	Host/ Indiv.	3,15 d	*Host proximity*	Distance from host shrub
2004	MBEST	Host/ Indiv.	3,15	*Host proximity*	Distance from host shrub
2006	FONR	1 m^2^ patch	6,25	*Mammalian herbivory*	Caged to exclude herbivores, open
2007	MBEST	Indiv.	5,10	*Insect seed predation*	*Bt* spray, water
2007	FONR “new”	25×25–cm plot, with 25 seeds each	1,10 e	*Fire suppression, Exotic species x Mammalian herbivory*	Charate, charcoal, or no soil amendment
2007	FONR “new”	25×25–cm plot, with 25 seeds each	3,30 e	*Fire suppression, Exotic species x Mammalian herbivory*	Cover: Highly Invaded, Moderately Invaded, “Scraped”

a Unit of replication. ‘Indiv.’ = Individual.

b ‘*x*’ indicates N with no blocks, ‘*x*,*y*’ indicates ‘blocks/treatments, N per block/treatment’.

c Total individuals from 17, 7 and 13 transects, respectively.

d One transect at FONR contained only 12 *C.r.l.* associated with *Artemisia californica*.

e Effects of charate were analyzed across 3 censuses by site and treatment, sites were not replicated, therefore N = 1, 10. Cover estimates were analyzed by site, therefore N = 3, 30.

### Inflorescences as a Proxy for Plant Fecundity

Number of inflorescences per plant was used as an indication of reproductive output in *C.r.l.* and was the dependent variable for most of the statistical tests reported below. To justify this proxy, we measured total number of inflorescences, pods per inflorescence, and total weight of all seeds for individuals in the *2006 Insect seed predation* experiment (n = 100; see below). Spearman rank-order correlations indicate significant positive associations between: number of inflorescences and the number of seed pods per individual (*r_s_* = 0.958, df = 98, *P* = 0.01) and number of pods and the total seed weight per individual (*r_s_* = 0.826, df = 98, *P* = 0.01).

Sets of flowers/seed pods separated by less than 4 cm of stem were considered a single inflorescence; this definition made estimates of the number of pods per inflorescence in our studies consistent, given the moderate range of differences in morphology among individuals. Inflorescences reported in all results include only those with filling pods (aborted flowers were ignored).

### Host Proximity (2004: FONR and MBEST)

To assess the influence of different host shrubs on the performance of *C.r.l.*, we compared growth and reproduction of *C.r.l.* individuals in close association with a single host shrub to individuals greater than 1 m from any woody perennial. *C.r.l.* were located randomly along three approximately 30–m transects per population. We tagged individuals rooted within 0.5 m of a single host species and at least 1 m away from all other shrub species. We also tagged individuals that were rooted at least 1 m from any woody perennial (controls). All tagged *C.r.l.* plants grew in a matrix of exotic annual grasses and forbs. We were only able to infer reduced host access in *C.r.l.* more than 1 m from woody perennials. However, at a distance of 1 m, host shrub roots are more scattered horizontally and the potential that some control individuals may have accessed hosts makes our results more conservative.

For both the FONR and MBEST populations, we assessed the performance of *C.r.l.* associated with California sagebrush, *Artemisia californica* (Less., Asteraceae), the only host shrub available in sufficient numbers at both sites. At MBEST we included coffeeberry, *Rhamnus californica* (Eschsch., Rhamnaceae) and at FONR we included California broom, *Lotus scoparius* ((Nutt.) Ottley, Fabaceae). We have physically documented *C.r.l.* haustorial attachment to the roots of these and other woody perennials at these sites (e.g. *Arctostaphylos* spp., Ericaceae).

At an initial census (15–20 July 2004), we tagged a total of 87 *C.r.l.* (FONR: 12 with *Artemisia*, 15 with *Lotus*, and 15 isolated; MBEST: 15 with *Artemisia*, 15 with *Rhamnus*, and 15 isolated). On 14–15 August 2004, we recorded height and the number of inflorescences for each *C.r.l.*. Data were log-transformed to meet assumptions of normality and analyzed using one-way ANOVAs (for each site) to determine if access to a host and/or host species influenced growth or inflorescence production. Tukey HSD post hoc tests were applied to determine differences in *C.r.l.* growth or reproduction by host.

### Mammalian Herbivory (2006: FONR)

To assess the impact of medium- to large-bodied mammalian herbivores on *C.r.l.*, on 22 April 2006 we selected 20, 1 m^2^ plots with patches of *C.r.l.*. Plots nearest one another were paired and one randomly chosen member of each pair was caged (2.1 m deer fence, 2.5 cm mesh); the other was left un-caged. Each plot had a 50 cm buffer (i.e. total area was 2×2 m). The mesh size of the fencing effectively excluded black-tailed deer (*Odocoileus hemionus*, Cervidae), brush rabbits (*Sylvilagus bachmani*, Leporidae), dusky-footed woodrats (*Neotoma fuscipes*, Cricetidae), California ground squirrels (*Spermophilus beecheyi*, Sciuridae), but would have allowed access to harvest mice (*Reithrodontomys* spp., Cricetidae), California voles (*Microtus californicus*, Cricetidae), brush mice (*Peromyscus boylii*, Cricetidae), California mice (*Peromyscus californicus*) and deer mice (*Peromyscus maniculatus*). Because fencing was not buried, pocket gophers (*Thomomys bottae*, Geomyidae) were able to access exclosures via tunnels.

Within each 1 m^2^ plot, 25 *C.r.l.* individuals were randomly chosen and measured at a final census (8 August 2006). Maximum height and width (height x width was used to estimate vertical area), the number of stems grazed by mammals (clear from sharp, angular cut ends of stems) and the number of inflorescences were recorded for each individual. Data for damaged stems were log-transformed to meet assumptions of normality, the remaining data met parametric assumptions; all were analyzed with paired *t*-tests.

Due to high natural mortality in both caged and open plots, dead individuals were recorded as such, and the nearest living previously unmeasured *C.r.l.* was measured as a replacement (unless the plot had fewer than 25 total *C.r.l.* individuals). To assess mortality we also counted the total number of *C.r.l.* in each 1 m^2^ plot. The proportion of *C.r.l.* surviving to the final census was arcsine transformed (angular transform) and analyzed with paired *t*-tests.

### Insect Seed Predation (2006: MBEST)

Senesced *C.r.l.* showed considerable seed predation by moth larvae (*Clepsis* sp., Tortricidae). On 3 August 2006 at the peak of flowering and prior to fruit development, 10 pairs of *C.r.l.* individuals were marked in each of five blocks (roughly 10×10 m areas) within the MBEST population (for a total of 100 *C.r.l.*). We then sprayed a randomly chosen member of each pair with *Bacillus thuringiensis* var. *kurstaki* (*Bt*, a lepidopteran specific insecticide) to determine the impact of moth larvae on *C.r.l.* seed set. The *Bt* spray consisted of Safer® Brand “Caterpillar Killer” mixed to specifications – 9.8 mL of concentrate per gallon (concentrate: 32 billion IU of potency per 3.8L). The other *C.r.l.* of each pair was sprayed with water as a procedural control. We sprayed each inflorescence and the entire plant until thoroughly wet. We reapplied treatments on August 4, 8, and 25.

By 29 August most *C.r.l.* individuals were senescing, so we recorded height and width and collected all individuals by cutting them at ground level. Insect damage was clear from ∼1 mm holes in flowers/pods, frass, silk webbing or the larvae themselves; pods showing this type of damage lost all seeds to the larvae. For each individual we recorded the total number of seed pods and the proportion of seed pods damaged per plant. Data concerning proportions of pods damaged were arcsine transformed and analyzed using two-way ANOVAs with block and treatment (e.g. *Bt*) as main effects.

### Exotic Species (2003: FONR and MBEST ; 2004: MBEST)

In July of 2003 and 2004, we set 30–m transects, 15 m apart in natural populations of *C.r.l.*. Five 1 m^2^ quadrats were distributed randomly along each 30–m transect. Within each quadrat we recorded *C.r.l.* presence/absence, exotic grass cover (predominately lodged exotic grasses), and litter depth in cm (average of three depths). In 2003, we censused 17 transects at MBEST and seven at FONR between 6 July and 25 August. In 2004, we censused 13 transects between 23 July and 10 August in the MBEST population. Cover data were arcsine transformed and analyzed with binary logistic regression. Nagelkerke *R* ^2^ was used; this version of the Cox and Snell *R* ^2^ extends the possible range of values to 1.

### Fire Suppression, Exotic Species X Mammalian Herbivory (2007: FONR “new”)

Some chaparral plants, particularly annuals, use chemical cues to time germination with post-fire conditions. Application of charate (charred and ground chaparral shrub stems) improves germination of many chaparral annuals [16] and can be an important management alternative in fire-adapted communities near human development. To test whether *C.r.l.* is sensitive to germination cues from charate we conducted this experiment at three, 20×14–m areas of FONR that lacked both *C.r.l.* and woody shrub species.

**Figure 1:**
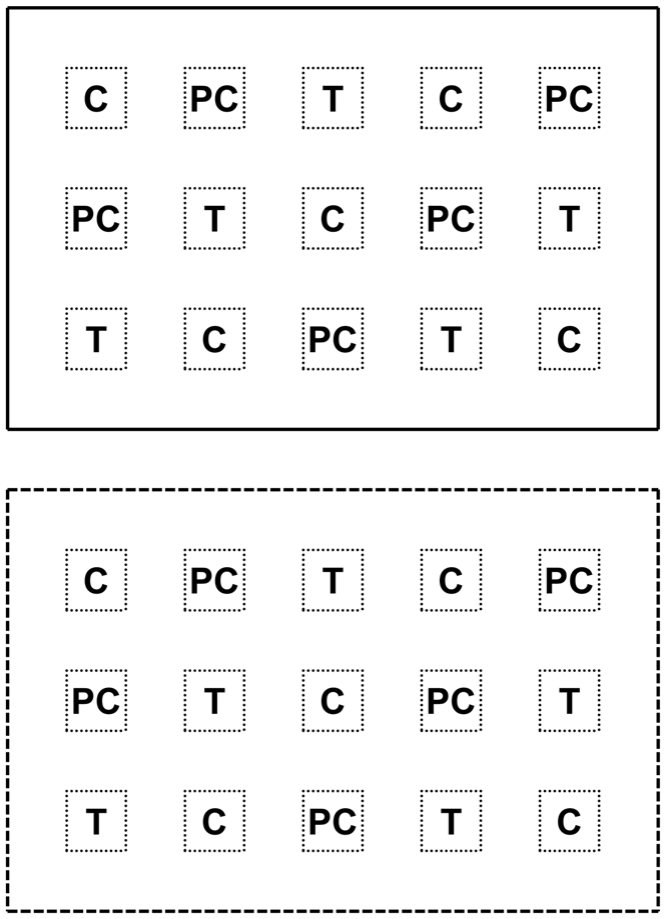
*2007 Fire suppression, Exotic species x Mammalian herbivory* design (not to scale). Solid lines indicate caged area, dashed lines indicate un-caged area, and dotted lines indicate 25×25–cm plots. Twenty-five *C.r.l.* seed were evenly sown in each plot and C, PC, and T indicate the distribution of each treatment (control, procedural control, or charate).

On 8 October 2006, stems (<1 cm diameter) from *Rhamnus californica* individuals at the FONR site were collected and baked in a drying oven at 175°C for 75 min (adapted from [22]). Baked stems were ground with a Wiley Mill (1 mm screen) to yield 1.25 kg of charate. As a procedural control for the simple addition of organic matter, the same amount of Kingsford ® brand regular charcoal (i.e. no lighter fluid) was ground to the same specifications. To provide a preliminary assessment of herbivore pressure, we erected cages on half of each of the three sites (10×14–m fenced areas, 2.1 m deer fence, 2.5cm mesh). We then marked 15, 25×25–cm plots inside and outside of each exclosure (Figure 1).

On 19 November 2006, 25 *C.r.l.* seeds (sorted with dissecting scopes to remove aborted seed) were sown in each plot for a total of 750 seeds per site. Prior to sowing, duff was clipped to 0.5cm height and removed. On 20 November 2006 five plots inside and outside each exclosure at these three sites received one of the following treatments: nothing (control; C), 25 g charcoal (procedural control; PC) or 25 g charate (treatment; T; Figure 1).

On 14 December 2006, we estimated percent cover of each of the following categories for each plot: exotic species, native species, bare ground, and litter (total = 100%). Cover data were arcsine transformed and analyzed using a Kruskal-Wallis nonparametric test. Tamhane's post hoc test was applied to determine which sites differed with regard to cover in each category.

We recorded *C.r.l.* germination and survivorship at monthly censuses: 24 February, 22 March, and 21 April. As the focus of this experiment was on recruitment limitations, the numbers of *C.r.l.* surviving to each census are presented. Data met parametric assumptions and repeated-measures ANOVA was used to determine the effect of charate over the course of the three censuses (within-subjects: census; between subjects: site and/or treatment).

The charate experiment was conducted at three sites at FONR with contrasting conditions: 1) *Highly Invaded*- dominated by invasive grass; few native species (e.g. *Lupinus* spp.), 2) *Moderately Invaded*- high density of invasives, but good representation of native annuals, 3) *“Scraped”*- topsoil previously removed from this site during military activity, exposing sandy, under-developed soil and resulting in low productivity and species diversity. Although site conditions were not replicated, this design allowed for a preliminary assessment of the interaction between several factors: herbivore pressure, soil development (*“Scraped”*), and invasive exotic plant species (esp. annual grasses). We report mean numbers of *C.r.l.* per plot (out of 25 seeds sown per plot) by site and exclosure.

## Results

### Host Proximity (2004: FONR and MBEST)

**Figure 2:**
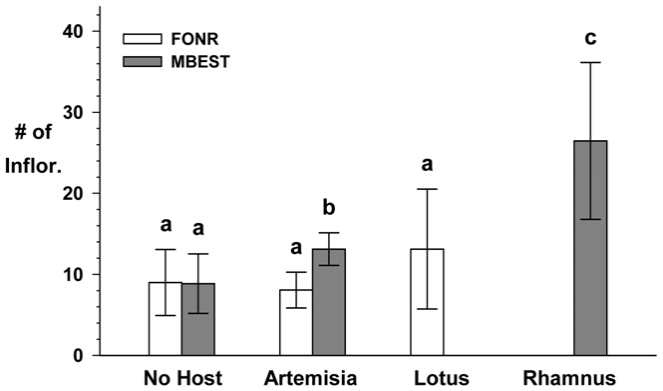
*2004 Host Proximity* results. Mean number of inflorescences for no-host (control) *C.r.l.*s and those within 0.5 m of perennial hosts: *A. californica*, *L. scoparius*, *R. californica*. Open bars = FONR, Filled bars = MBEST. Error bars are 95% C.I.s. Bars not sharing lowercase letters are significantly different within sites. Note: Difference between *A. californica* and control at MBEST is only marginally significant. Also, raw data are shown here; however, analyses were performed on log transformed data.

At FONR, the number of inflorescences did not differ among *C.r.l* plants growing next to *Lotus scoparius*, *Artemisia californica,* or controls (*F*_2,39_ = 0.302, *P* = 0.741; Figure 2). At MBEST, in contrast, *C.r.l.* growing near *A. californica* and those near *Rhamnus californica* produced significantly more inflorescences than controls (*F*_2,42_ = 12.793, *P*<0.001; see Table 2 for post hoc tests). On average at MBEST, *C.r.l.* growing near *R. californica* produced three times as many inflorescences as those *C.r.l.* without hosts and twice as many as those near *A. californica* (although at *P* = 0.05 this difference was marginal).

**Table 2:** *2004 Host proximity*: Tukey HSD post hoc tests for differences in natural log of the number of inflorescences by host (sites were analyzed separately).

Site	(I) Host	(J) Host	Mean Diff. (I-J)	*P* a
FONR	Control	*Lotus*	−0.2065	0.732
		*Artemisia*	−0.0576	0.978
FONR	*Artemisia*	Control	0.0576	0.978
		*Lotus*	−0.1489	0.865
FONR	*Lotus*	Control	0.2065	0.732
		*Artemisia*	0.1489	0.865
MBEST	Control	*Rhamnus*	−1.0603	**<0.001**
		*Artemisia*	−0.5515	**0.031**
MBEST	*Artemisia*	Control	0.5515	**0.031**
		*Rhamnus*	−0.5088	**0.050** b
MBEST	*Rhamnus*	Control	1.0603	**<0.001**
		*Artemisia*	0.5088	**0.050** b

a **Bold** indicates mean difference is significant at the 0.05 level.

b Mean difference is marginally significant at the 0.05 level.

Differences in plant height relative to host proximity were more pronounced than those for inflorescences, but with a similar pattern. In contrast to the inflorescence results, plant heights at FONR differed among *C.r.l* plants growing next to *L. scoparius*, *A. californica,* and controls (*F*_2,39_ = 3.492, *P* = 0.04; see Table 3 for post hoc tests); post hoc tests show that differences in *C.r.l.* growth were restricted to those growing near *L. scoparius*. At MBEST, *C.r.l.* near *R. californica* grew significantly taller than either controls or those growing near *A. californica*; control *C.r.l.* did not differ from those growing near *A. californica* (*F*_2,42_ = 11.421, *P*<0.001; see Table 3).

**Table 3:** *2004 Host proximity*: Tukey HSD post hoc tests for differences in natural log of height by host (sites were analyzed separately).

Site	(I) Host	(J) Host	Mean Diff. (I-J)	*P* a
FONR	Control	*Lotus*	−4.7267	**0.039**
		*Artemisia*	−1.1483	0.830
FONR	*Artemisia*	Control	1.1483	0.830
		*Lotus*	−3.5783	0.177
FONR	*Lotus*	Control	4.7267	**0.039**
		*Artemisia*	3.5783	0.177
MBEST	Control	*Rhamnus*	−15.5867	**<0.001**
		*Artemisia*	−6.9267	0.098
MBEST	*Artemisia*	Control	6.9267	0.098
		*Rhamnus*	−8.6600	**0.030**
MBEST	*Rhamnus*	Control	15.5867	**<0.001**
		*Artemisia*	8.6600	**0.030**

a **Bold** indicates mean difference is significant at the 0.05 level.

### Mammalian Herbivory (2006: FONR)

**Figure 3:**
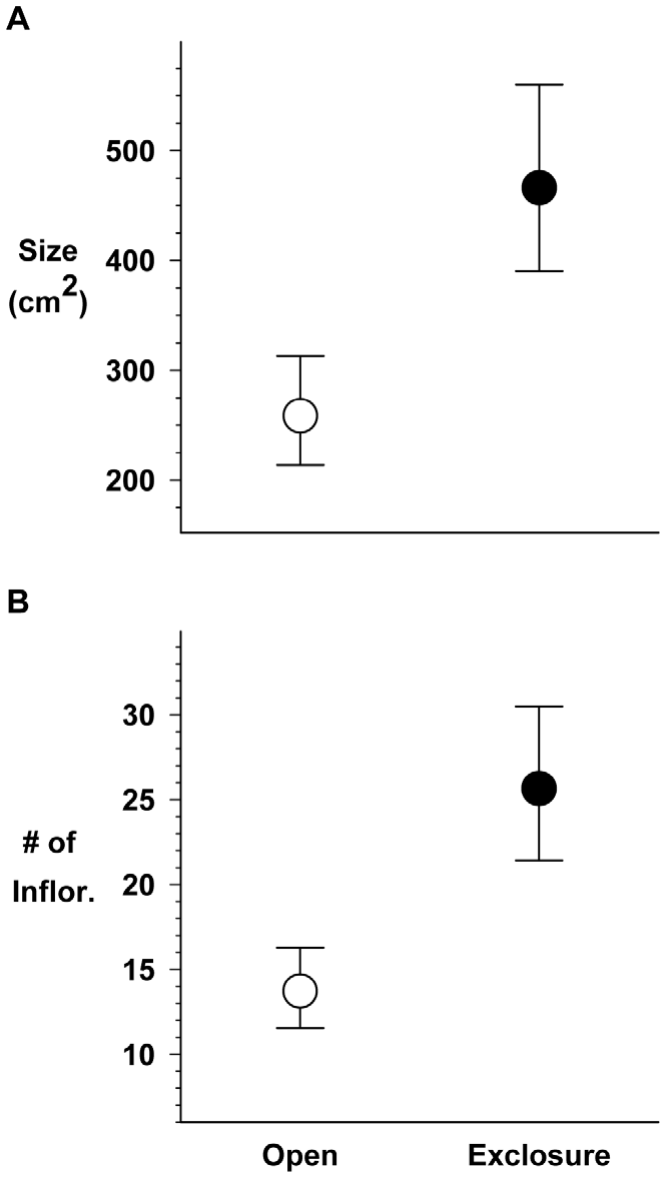
*2006 Mammalian Herbivory* results. **A** Size (height×width: cm^2^) of open (exposed) vs. exclosure individuals. **B** Number of inflorescences produced by open vs. exclosure individuals. Error bars are 95% C.I.s.

Several plots (both fenced and unfenced) were invaded by pocket gophers that killed *C.r.l.* through both tunneling and mound production. Plots with more than 5% cover of pocket gopher mounds were dropped from the analysis, along with their paired plots (four pairs excluded). Paired *t*-tests of the remaining six paired plots showed that *C.r.l.* were on average 2.1 times larger (cm^2^) with 2.3 times the number of inflorescences in caged than in open plots (cm^2^: *t* = −3.514, df = 5, *P* = 0.017; total inflorescences: *t* = −4.445, df = 5, *P* = 0.007; Figure 3A and B).

We did not formally control for cage effects in our design, however, caging would be expected to increase inter-/intraspecific competition, yielding smaller and less productive individuals; the opposite occurred. Mortality was not significantly greater in caged vs. open plots (Mortality: *t* = −0.828, df = 5, *P* = 0.446). Finally, differences in mean size and number of inflorescences was observed as a direct result of grazing by mammals as the number of clipped stems was greater in caged than in open plots (Clipped Stems: *t* = 2.687, df = 5, *P* = 0.043).

### Insect Seed Predation (2006: MBEST)

The *Bt* solution reduced the proportion of *C.r.l.* pods damaged by insects by 12.5% (39.6%±0.10 SE vs. 27.1%±0.12 SE damaged pods; *F*_1,4_ = 8.424, *P* = 0.005), there were no significant differences among blocks (*F*_1,4_ = 1.089, *P* = 0.367) and no block x treatment interaction (*F*_1,4_ = 2.295, *P* = 0.065). This translated directly to increased seed production because infested pods lost all seed to the larvae and there was no difference between control and *Bt* plants in the number of pods per plant (mean: 68.3±7.2 pods/plant; Control vs. *Bt* plants: *F*_1,4_ = 0.107, *P* = 0.744).

### Exotic Species (2003: FONR and MBEST; 2004: MBEST)

At MBEST, *C.r.l.* presence was negatively correlated with litter depth in both years and with exotic grass cover in 2003 (Table 4). At FONR, which was only censused in 2003, *C.r.l.* presence was not correlated with either litter depth or grass cover. Significant correlations between *C.r.l.* presence and either factor had relatively low *R* ^2^ values, which likely reflects the combined influence of a number of other factors not measured here, including host availability and soil characteristics.

**Table 4:** *2003/04 Exotic species*: Results of binary logistic regression between *C.r.l.* presence and average leaf litter depth or arcsine percent grass cover.

Factor	Site	Year	n	Constant a	Slope	*R* ^2^	*P*
Litter	MBEST	2003	85	0.388	−0.810	0.139	**0.022**
	MBEST	2004	65	0.518	−0.125	0.146	**0.024**
	FONR	2003	35	0.275	−0.035	0.026	0.419
Grass	MBEST	2003	85	0.868	−0.045	0.172	**0.003**
	MBEST	2004	65	−0.561	0.005	0.002	0.746
	FONR	2003	35	−0.574	0.020	0.029	0.391

a Equation: **ln [p/(1-p)]** ***C.r.l.*** **presence** = ***α*** **+** ***β*** **(litter/grass)**, where **p** = probability of *C.r.l.* presence, ***α*** = constant and ***β*** = slope. Nagelkerke ***R*** **^2^**, significant ***P***-values in **bold**; df = 1.

### Fire Suppression, Exotic Species X Mammalian Herbivory (2007: FONR “new”)

**Figure 4:**
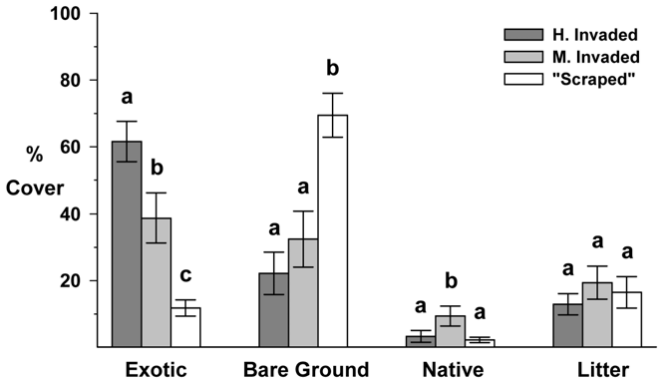
*2007 Fire suppression, Exotic species x Mammalian herbivory* cover estimates. Mean percent cover by category for each “new” site: “Scraped” (no fill), Moderately Invaded (light gray fill) and Highly Invaded (dark gray fill). Error bars are 95% C.I.s. Bars not sharing lowercase letters are significantly different within cover categories.

Cover data support our Highly Invaded, Moderately Invaded, and “Scraped” site characterizations (see Table 5 and Figure 4). All three sites differed with regard to exotic species cover (*X* ^2^ = 56.91, df = 2, *P*<0.001). The “Scraped” site had significantly more bare ground than the other two sites, which did not differ from each other (*X* ^2^ = 44.20, df = 2, *P*<0.001). The Moderately Invaded site had significantly greater native species cover than the other two sites, which did not differ from each other (*X* ^2^ = 31.03, df = 2, *P*<0.001). Finally, due to our clipping and duff removal, there were no significant differences in litter cover among sites (*X* ^2^ = 3.34, df = 2, *P*<0.188).

**Table 5:** *2007 Fire suppression, Exotic species x Mammalian herbivory*: Tamhane's post hoc tests for differences in arcsine percent cover of exotic species, bare ground, native species, and litter by site.

Cover Type	(I) Site	(J) Site	Mean Diff. (I-J)	*P* a
Exotic Spp.	H. Invaded	M. Invaded	14.18226	**<0.001**
		“Scraped”	32.48098	**<0.001**
Exotic Spp.	M. Invaded	H. Invaded	−14.18226	**<0.001**
		“Scraped”	18.29872	**<0.001**
Exotic Spp.	“Scraped”	H. Invaded	−32.48098	**<0.001**
		M. Invaded	−18.29872	**<0.001**
Bare Ground	H. Invaded	M. Invaded	−7.34022	**0.154**
		“Scraped”	−31.17642	**<0.001**
Bare Ground	M. Invaded	H. Invaded	7.34022	0.154
		“Scraped”	−23.83620	**<0.001**
Bare Ground	“Scraped”	H. Invaded	31.17642	**<0.001**
		M. Invaded	23.83620	**<0.001**
Native Spp.	H. Invaded	M. Invaded	−7.68977	**<0.001**
		“Scraped”	1.36915	0.670
Native Spp.	M. Invaded	H. Invaded	7.68977	**<0.001**
		“Scraped”	9.05892	**<0.001**
Native Spp.	“Scraped”	H. Invaded	−1.36915	0.670
		M. Invaded	−9.05892	**<0.001**
Litter	H. Invaded	M. Invaded	−4.64700	0.128
		“Scraped”	−2.50744	0.589
Litter	M. Invaded	H. Invaded	4.64700	0.128
		“Scraped”	2.13957	0.787
Litter	“Scraped”	H. Invaded	2.50744	0.589
		M. Invaded	−2.13957	0.787

a **Bold** indicates mean difference is significant at the 0.05 level.

**Figure 5:**
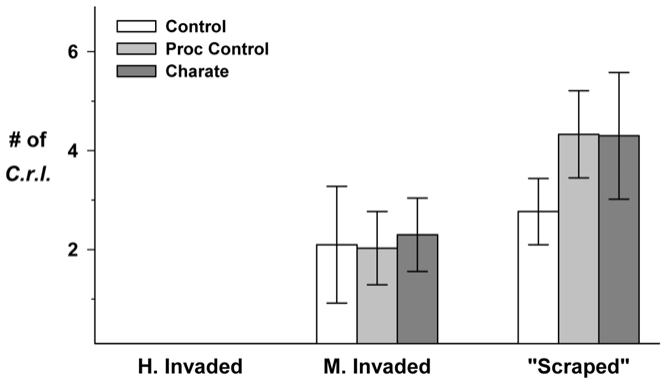
*2007 Fire suppression, Exotic species x Mammalian herbivory* results. Mean number of *C.r.l.* individuals per plot at each site (Highly Invaded, Moderately Invaded and “Scraped”) under charate treatments: control (no fill), procedural control (light gray fill), and charate (dark gray fill). Error bars are 95% C.I.s.

The Highly Invaded area was dropped from statistical analyses as there was no recruitment of *C.r.l.* in these plots (out of 750 seeds sown). At the two locations that did experience some recruitment (Moderately Invaded and “Scraped”), charate application did not significantly increase germination (RMANOVA charate: *F*_2,54_ = 1.031, *P* = 0.363; Figure 5). However, the data (Table 6) suggest that the addition of organic matter (charate or charcoal) may have improved *C.r.l.* recruitment at the “Scraped,” but not the Moderately Invaded site.

**Table 6:** *2007 Fire suppression, Exotic species x Mammalian herbivory*: Mean number of *C.r.l.* per plot surviving to the final census at each site (FONR 2007) by treatment; “*n*” for each row indicates plot sub-samples within each site (see Figure 1).

		Moderately Invaded	“Scraped”
Treatment	*n*	# *C.r.l.*/plot ± SE	# *C.r.l.*/plot ± SE
Control (C)	10	2.10±0.60	2.77±0.34
Charcoal (PC)	10	2.03±0.38	4.33±0.45
Charate (T)	10	2.30±0.38	4.30±0.65
Open	15	2.51±0.35	3.09±0.36
Exclosure	15	1.78±0.39	4.51±0.45
Total	30	2.14±0.26	3.80±0.29

**Figure 6:**
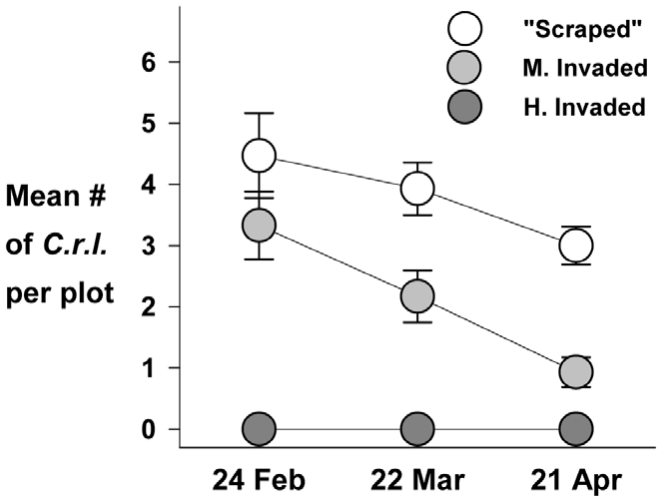
*2007 Fire suppression, Exotic species x Mammalian herbivory* results. Mean number of *C.r.l.* individuals per plot at each area through 2007 season: “Scraped” (no fill), Moderately Invaded (light gray fill) and Highly Invaded (dark gray fill). Error bars are SE for each census.

Although the three competitive environments were not replicated, it is worth noting that there was no recruitment of *C.r.l.* at the Highly Invaded area. Independent of any effects of exclosures or charate, germination and recruitment success were greater at the “Scraped” area, despite the lack of soil development, than at the Moderately Invaded area (Table 6) and survivorship through the season remained higher at the “Scraped” site than at the Moderately Invaded site (Figure 6).

## Discussion

*C.r.l.* reproduction is enormously variable from year-to-year and site-to-site, with seed production varying from zero to as many as ten thousand seeds per plant (see supporting information Figure S3). The boom years of seed production, however, do not necessarily mean success as seed viability and germination rates appear to be extremely low – from zero germination (out of 750 seeds) in the Highly Invaded site to 17.8%±2.77 SE mean germination in the scraped site (under laboratory conditions we found even lower seed viability: 2.8%±0.89 SE; see supporting information Site and Species Description S1). This combination of potentially high rates of reproduction with rare success means that the recovery of this endangered California species will require mitigating the factors limiting *C.r.l.* recruitment and, by extension, the establishment of new populations.

Our field studies show that host availability and herbivory, among several other factors, impact *C.r.l.* growth and reproduction at FONR and MBEST. Although we cannot be certain that the control *C.r.l.* had no access to host shrubs, close proximity to *Rhamnus*, in particular, tended to triple the reproduction of *C.r.l.* compared to *C.r.l.* without nearby hosts (Figure 2); proximity to *Artemisia californica* had a similar effect, but it was not quite as dramatic. It is notable that hosts were found to significantly improve *C.r.l.* reproduction at MBEST, but not at FONR. As discussed below, it is possible that mammalian herbivory could modify the benefits of host proximity. Even *C.r.l.* that have access to hosts and survive or tolerate mammalian herbivory may lose up to 40% of their seeds to *Clepsis* larvae, which could limit *C.r.l*. 's ability to replenish the seed bank for the following growing season. The results of the charate experiment, in contrast, suggest that *C.r.l.* germination, at least, does not require the chemical cues that would result from fire.

Combined conclusions of the two studies of exotic species competition and litter deposition (2003/04 *Exotic species*, 2007 *Fire suppression, Exotic species x Mammalian herbivory*) suggest that these factors might be important in exacerbating other threats to *C.r.l.* populations; especially considering the contrasting results from one year to the next and between sites. Given that the three contrasting sites for the 2007 study were not replicated and that there are likely other factors involved, the mechanism of complete germination suppression in the Highly Invaded site is unclear. Although, litter and germinating plants were clipped and removed from the plots when *C.r.l.* were sown, re-growth and recruitment of exotic plants was very rapid in the Highly and Moderately Invaded sites (comprising approximately 62% and 39% cover for each site, respectively, in less than a month; see Figure 4), so it is possible that any germinants at the Highly Invaded site may have died during the interval between censuses. The relatively few *C.r.l.* that manage to access hosts benefit from reduced summer competition, however, *C.r.l.* competes as an annual among the rapidly growing exotics from its relatively late germination in February through June. The role of exotic species now warrants more finely tuned manipulative experiments to investigate the influence of litter on germination and direct competition from exotic species on growth.

Until further research is done on exotic species and interactions among the threats to *C.r.l.*, we can provisionally recommend several management principles for *C.r.l.*. First, with so few natural populations of *C.r.l.*, it is critical that additional populations be established to reduce extinction risk. This is a particularly urgent need as the MBEST site (with more *C.r.l.*) is not currently protected by the network of habitat reserves. *C.r.l.* seed should, therefore, be collected in “good years,” and reserve managers might consider targeted applications of *Bt* to reduce seed predation and promote individual fecundity in “bad years”. Collecting seeds is important for the establishment of new *C.r.l.* populations because its seeds have no apparent secondary dispersal ability and most fall a short distance from the parent plant – much reducing the likelihood of natural establishment, especially given the fragmentation of California coastal habitats. Tentatively, we recommend attempts to manage invasive annual species using a range of techniques and across several sites in FONR, followed by studies to determine best practices for establishment of new *C.r.l.* populations in these areas from seed. If seeds and other resources are limited, we would suggest that these studies focus on establishment near *Rhamnus californica*, as being near it, in particular, appears to improve fecundity.

In the absence of the studies reported here and additional experiments, management efforts aimed at *C.r.l.* are well-intentioned guesses. There is still much we do not know about longer term and broader spatial scale population processes at work in this system, but the studies we report can suggest hypotheses to test more explicitly. For example, while caging is an obvious management tool, our 2006 mammalian herbivory exclosures showed no negative cage effects in natural populations, whereas our attempts to establish new populations (2007 Fire/Exotics/Herbivory) suggest this may not apply outside of these naturally occurring patches (see also supporting information site photos Figure S4).

The next step in our research is to better explore the role of exotic species and to pursue factorial experiments that simultaneously manipulate multiple limiting factors. Extant *C.r.l.* populations are often found in open scrub where they benefit from lower competition, while maintaining access to host shrubs (Figure S5 and S6). However, in such patchy shrub habitats *C.r.l.* are subject to exotic-species invasion (relative to closed canopy shrub habitats; [23]). Furthermore, we do not know how the benefits of host proximity relate to the potential for greater herbivore pressure from small mammals sheltering in shrubs (see [24]). Ironically, the lack of reserve protection for mammals at MBEST may help explain the consistent differences between MBEST and FONR in the abundance and performance of *C.r.l.*. Finally, there are several categories of factors that we were not able to assess, particularly the limitations imposed by soil characteristics, such as pH and moisture. The influence of these factors would be amenable to investigation in the same manner as our current set of studies.

The total budget for the research reported here was under $12000, including stipends and conference travel for the three undergraduate coauthors of this paper. This highlights the utility of even small-scale quantitative assessments of threats to endangered species, when a modest investment can yield some clear experimental evidence and otherwise point to gaps in our current understanding [3].

While there are many challenges to recovering *C.r.l.*, the fact that individual plants can produce huge numbers of seeds suggests that this species has an excellent chance of survival if we implement an evidence based recovery program.

## Supporting Information

Site and Species Description S1 (0.04 MB DOC)

Figure S1 Photo of *C.r.l.* haustoria. Four attachment points of *C.r.l.* haustoria (lighter root) on root of *Arctostaphylos* sp. (manzanita- a woody shrub, Ericaceae).
(2.11 MB TIF)

Figure S2 Map of study sites. Inset displays Santa Cruz and Monterey County (blue area in California map) and FONR (blue area in Monterey County). Extent of aerial view is boxed in inset. Eastern portion of FONR is outlined in black in aerial view. White-line polygons trace the extent of two natural populations **A** FONR and **B** MBEST. Three “new” sites are indicated as follows: **C** Highly Invaded, **D** Moderately Invaded and **E** “Scraped.” These five sites (two natural and three “new”) lie roughly within latitude 36°40′N to 36°41′N and longitude 121°45′W to 121°46′W.
(4.17 MB TIF)

Figure S3 Plot of *C.r.l.* surveys and precipitation at FONR and MBEST. Surveys were conducted by UC FONR management in late spring 2001 - 2008 and include the total number of individuals in each population. Climate data were compiled by Carl Thormeyer from his residence in Marina, CA.
(7.95 MB TIF)

Figure S4 Photo from outside of exclosure at Moderately Invaded site. Note the difference in species composition: darker stems on the left - within the exclosure - are *Rumex acetosella* (Polygonaceae), an exotic species.
(10.19 MB TIF)

Figure S5 FONR Natural Population. Photo of maritime chaparral and oak woodland ecotone of the FONR natural population. Note caged and un-caged plots (middle ground) for *2006 Mammalian herbivory*. Corners of plots marked with blue flags.
(6.62 MB TIF)

Figure S6 MBEST Natural Population. Photo of open maritime chaparral habitat of the MBEST natural population. Note *C.r.l.* near base of shrubs.
(9.80 MB TIF)
