# Supplementary material for: Using Small-Scale Studies to Prioritize Threats and Guide Recovery of a Rare Hemiparasitic Plant: Cordylanthus rigidus ssp. littoralis
Source: PLoS One. 2010 Jan 26;5(1):e8892. doi: 10.1371/journal.pone.0008892 (PMC2811196; doi:10.1371/journal.pone.0008892)
Supplement: Site and Species Description S1 — (0.04 MB DOC) [file pone.0008892.s001.doc]

**Supporting Information**

Site and Species Description S1.

*Cordylanthus rigidus* ssp. *littoralis* is found on the frequently disturbed sandy soils of stabilized dunes in closed-cone pine forest, cismontane woodland, or maritime chaparral communities. Though the Jepson Manual (Hickman 1993) describes *C.r.l.* as having 5-8 flowered inflorescences, we found a mean of only 2.36 ± 0.06 SE fruits (pods) per inflorescence in the populations we studied. The total number of infructescences per individual ranged widely- from less than 10 to over 1000 when herbivores were excluded. Seed pods are ~1cm in length and we found that each pod included 6 – 140 ~0.5mm seeds (mean: 19.38 ± 1.86 SE). In 2006, seed viability tests were conducted in 17 petri dishes with filter paper and 25 seeds (sorted with dissecting scopes to remove aborted seed). After one month (1 August – 2 September) only 12 of 425 seeds had germinated, with a mean germination of 2.82% ± 0.892 SE.
