# Supplementary figures and images for: Using Small-Scale Studies to Prioritize Threats and Guide Recovery of a Rare Hemiparasitic Plant: Cordylanthus rigidus ssp. littoralis
Source: PLoS One. 2010 Jan 26;5(1):e8892. doi: 10.1371/journal.pone.0008892 (PMC2811196; doi:10.1371/journal.pone.0008892)

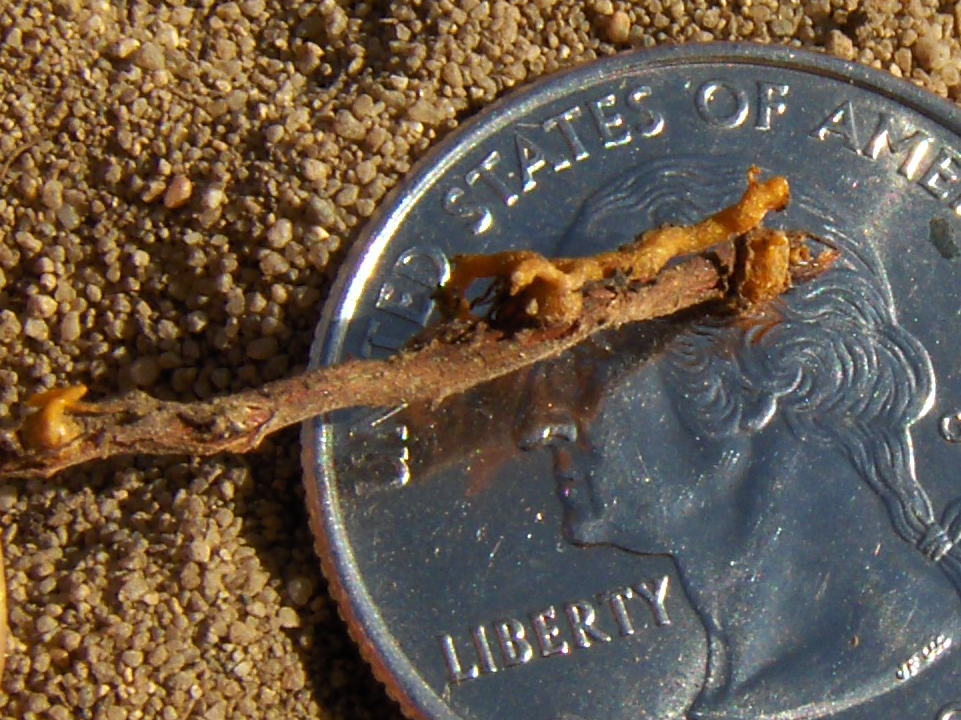

Supplement: Figure S1 — Photo of C.r.l. haustoria. Four attachment points of C.r.l. haustoria (lighter root) on root of Arctostaphylos sp. (manzanita- a woody shrub, Ericaceae). (2.11 MB TIF) [file pone.0008892.s002.tif]

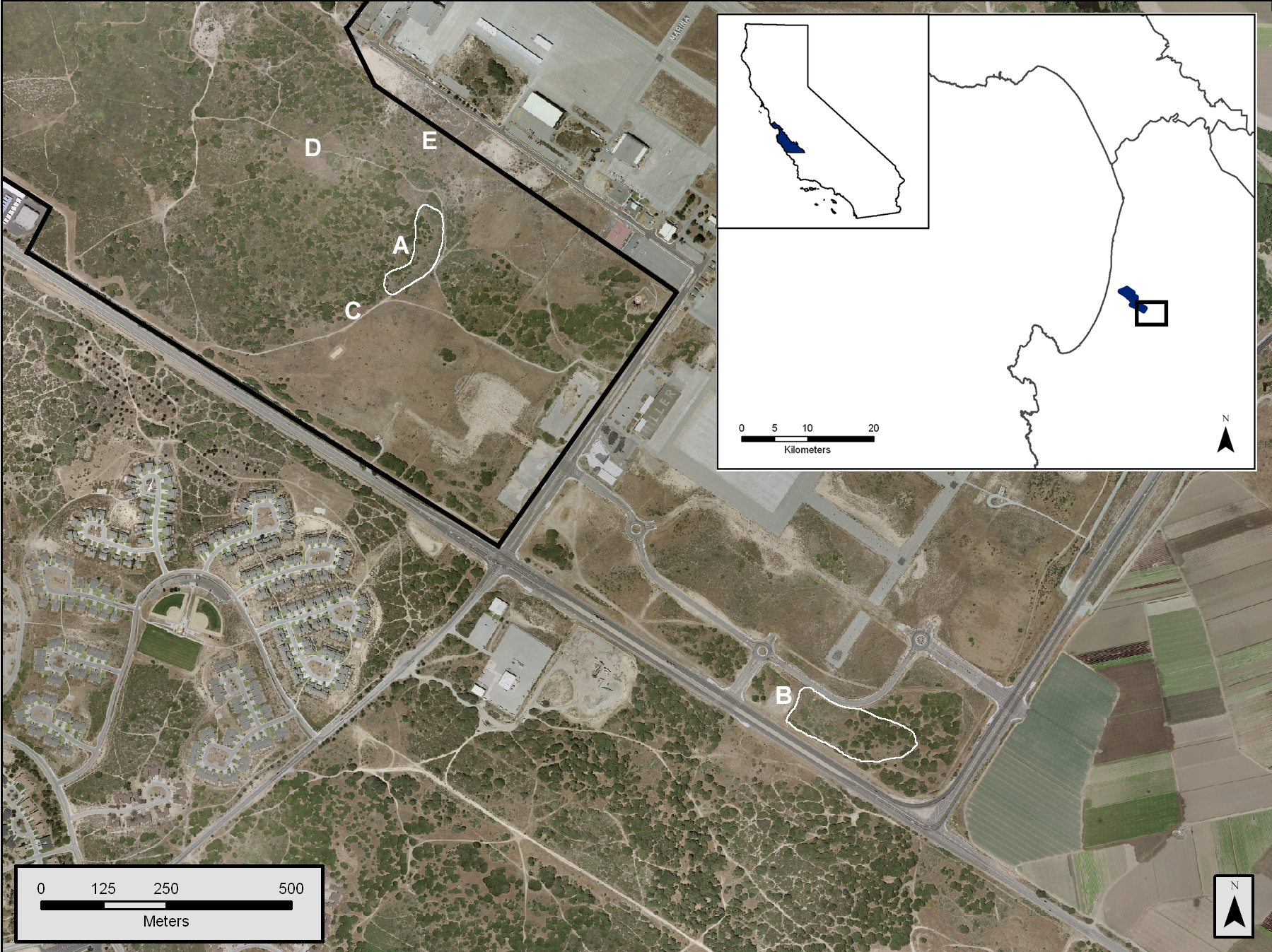

Supplement: Figure S2 — Map of study sites. Inset displays Santa Cruz and Monterey County (blue area in California map) and FONR (blue area in Monterey County). Extent of aerial view is boxed in inset. Eastern portion of FONR is outlined in black in aerial view. White-line polygons trace the extent of two natural populations A FONR and B MBEST. Three “new” sites are indicated as follows: C Highly Invaded, D Moderately Invaded and E “Scraped.” These five sites (two natural and three “new”) lie roughly within latitude 36°40′N to 36°41′N and longitude 121°45′W to 121°46′W. (4.17 MB TIF) [file pone.0008892.s003.tif]

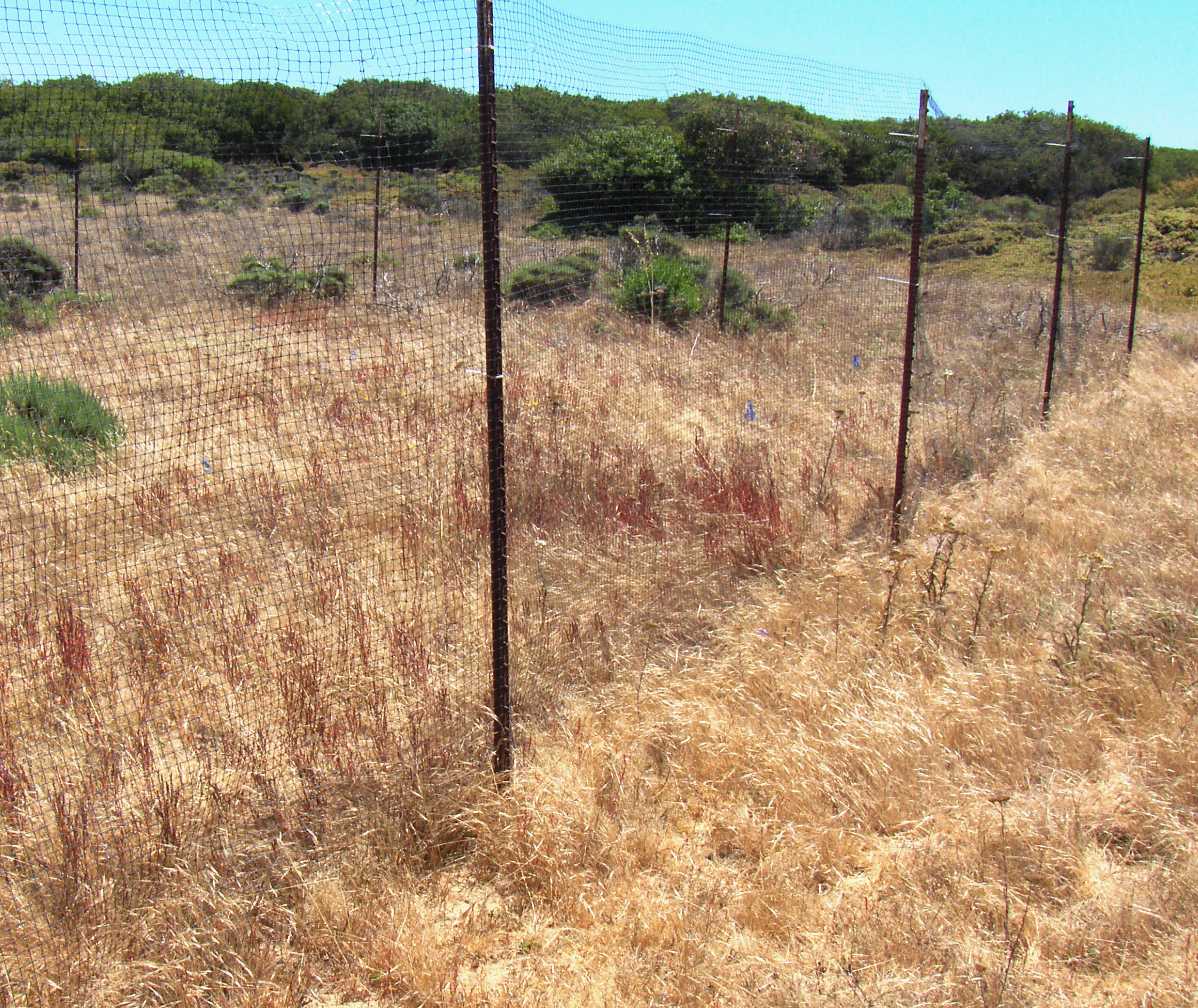

Supplement: Figure S4 — Photo from outside of exclosure at Moderately Invaded site. Note the difference in species composition: darker stems on the left - within the exclosure - are Rumex acetosella (Polygonaceae), an exotic species. (10.19 MB TIF) [file pone.0008892.s005.tif]

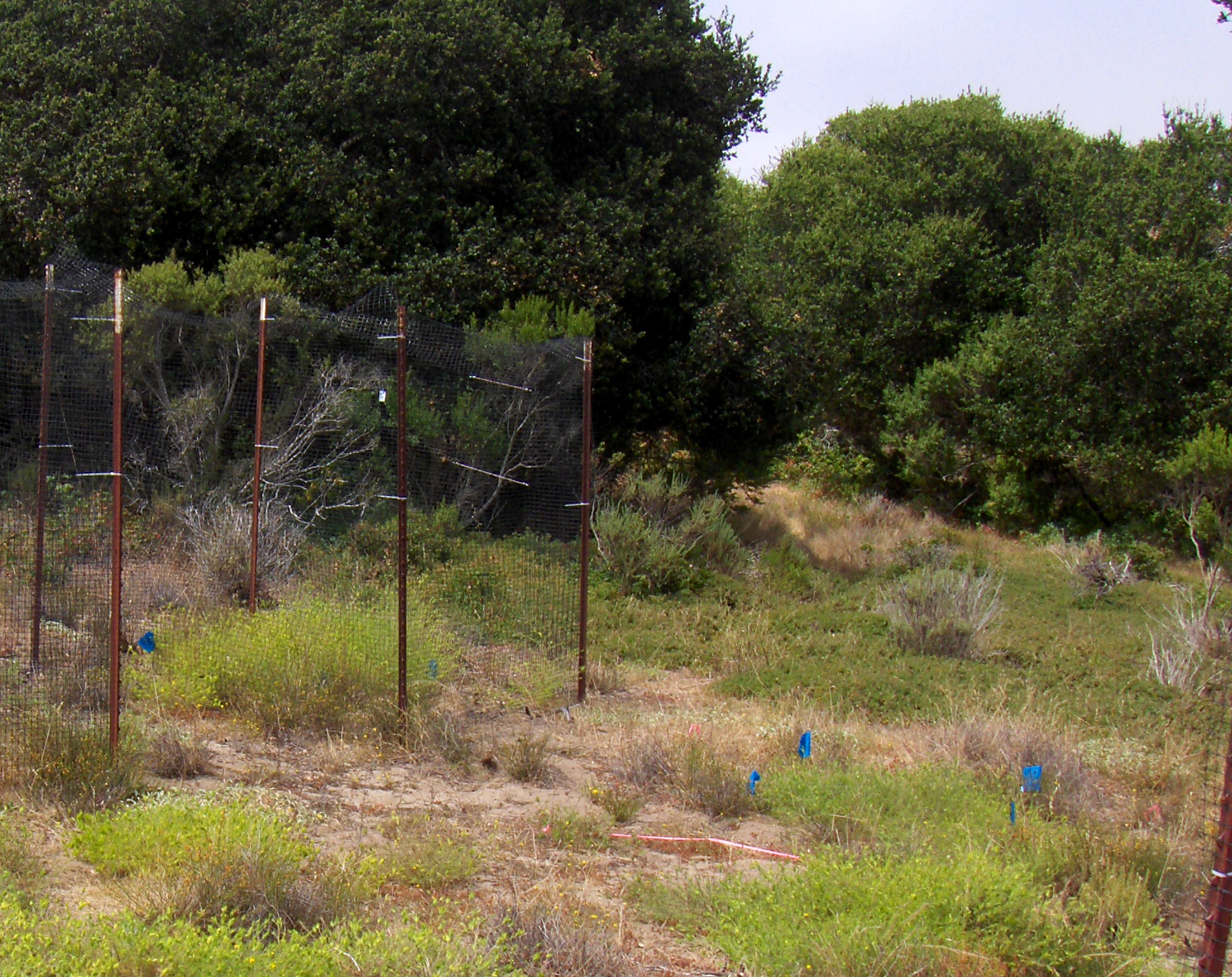

Supplement: Figure S5 — FONR Natural Population. Photo of maritime chaparral and oak woodland ecotone of the FONR natural population. Note caged and un-caged plots (middle ground) for 2006 Mammalian herbivory. Corners of plots marked with blue flags. (6.62 MB TIF) [file pone.0008892.s006.tif]

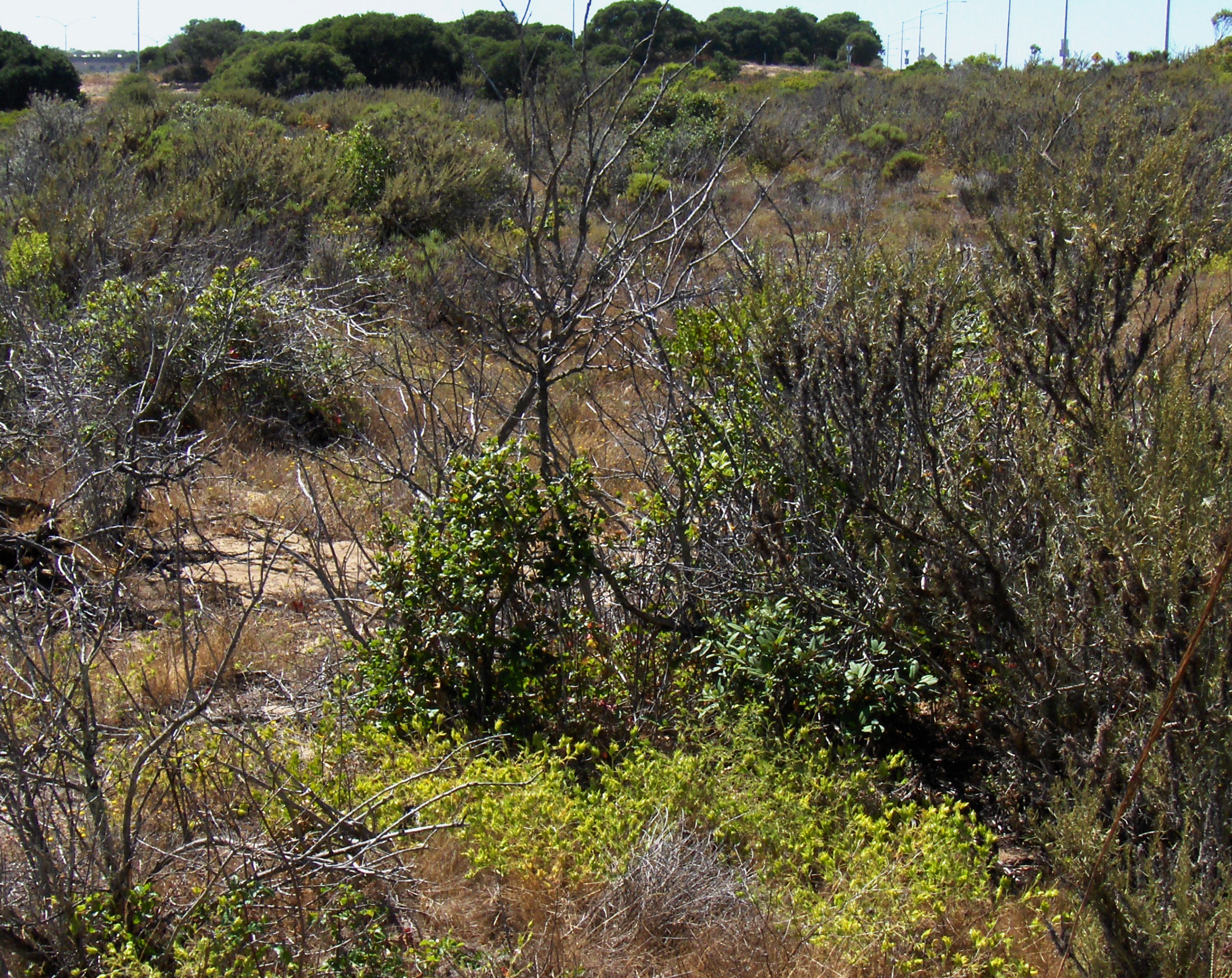

Supplement: Figure S6 — MBEST Natural Population. Photo of open maritime chaparral habitat of the MBEST natural population. Note C.r.l. near base of shrubs. (9.80 MB TIF) [file pone.0008892.s007.tif]
